# raxtax: a *k*-mer-based non-Bayesian taxonomic classifier

**DOI:** 10.1093/bioinformatics/btaf620

**Published:** 2025-11-19

**Authors:** Noah A Wahl, Georgios Koutsovoulos, Ben Bettisworth, Alexandros Stamatakis

**Affiliations:** Biodiversity Computing Group, Institute of Computer Science, Foundation for Research and Technology Hellas, N. Plastira 100, Heraklion, Crete, 70013, Greece; Biodiversity Computing Group, Institute of Computer Science, Foundation for Research and Technology Hellas, N. Plastira 100, Heraklion, Crete, 70013, Greece; Biodiversity Computing Group, Institute of Computer Science, Foundation for Research and Technology Hellas, N. Plastira 100, Heraklion, Crete, 70013, Greece; Biodiversity Computing Group, Institute of Computer Science, Foundation for Research and Technology Hellas, N. Plastira 100, Heraklion, Crete, 70013, Greece; Computational Molecular Evolution Group, Heidelberg Institute for Theoretical Studies, Schloß-Wolfsbrunnenweg 35, Heidelberg, Baden-Württemberg, 69118, Germany; Institute of Theoretical Informatics, Karlsruhe Institute of Technology, Kaiserstraße 12, Karlsruhe, Baden-Württemberg, 76131, Germany

## Abstract

**Motivation:**

Taxonomic classification in biodiversity studies is the process of assigning the anonymous sequences of a marker gene (barcode) or whole genomes (metagenomics) to a specific lineage using a reference database that contains named sequences in a known taxonomy. This classification is important for assessing the diversity of biological systems. Taxonomic classification faces two main challenges: first, accuracy is critical as errors can propagate to downstream analysis results; and second, the classification time requirements can limit study size and study design, in particular when considering the constantly growing reference databases. To address these two challenges, we introduce raxtax, an efficient, novel taxonomic classification tool for barcodes that uses common *k*-mers between all pairs of query and reference sequences. We also introduce two novel uncertainty scores which take into account the fundamental biases of reference databases.

**Results:**

We validate raxtax on three widely-used empirical reference databases and show that it is 2.7–100 times faster than competing state-of-the-art tools on the largest database while being equally accurate. In particular, raxtax exhibits increasing speedups with growing query and reference sequence numbers compared to existing tools (for 100 000 and 1 000 000 query and reference sequences overall, it is 1.3 and 2.9 times faster, respectively), and therefore alleviates the taxonomic classification scalability challenge.

**Availability and implementation:**

raxtax is available at https://github.com/noahares/raxtax under a CC-NC-BY-SA license. The scripts and summary metrics used in our analyses are available at https://github.com/noahares/raxtax_paper_scripts. The source code, sequence data, and summarized results of the analyses are available at https://doi.org/10.5281/zenodo.15057027.

## 1 Introduction

Biodiversity researchers frequently need to address the question: Which species are present in my sample? A common solution consists in identifying and subsequently sequencing a well-conserved region of the genome which is present in all organisms under study (Hebert *et al.* 2003b, Ward *et al.* 2005, Schoch *et al.* 2012). Such regions, known as barcodes (Hebert *et al.* 2003a), are then used to identify species. The ribosomal 16S gene, the cytochrome oxidase 1 (COX1), and the internal transcribed spacer (ITS) regions are examples of frequently used barcodes in distinct regions of the tree of life (see, e.g. Janssen 2006, Elbrecht *et al.* 2016, Yang *et al.* 2018). As using barcodes for DNA-based species identification constitutes a routine analysis task, there exist several widely-used taxonomic classification tools, such as SINTAX (Edgar 2016), IDTAXA (Murali *et al.* 2018), the RDP Naive Bayesian classifier (RDP) (Wang *et al.* 2007), and BayesANT (Zito *et al.* 2023). These highly cited tools deploy distinct algorithmic approaches to determine the species that are present in a sample.

The major design and one major quality criterions for any taxonomic classification tool are: assign sequences quickly and correctly. Species identification accuracy is critical, as it typically constitutes the first step in biodiversity analyses. Therefore, errors are likely to be propagated to downstream analyses and results. However, we are in the midst of the next generation sequencing data avalanche which is being further intensified by an increasing number of biodiversity field studies (Liu *et al.* 2011, La Salle *et al.* 2016). The amount of data being generated has outpaced Moore’s law for the last decade (Wetterstrand 2019). Hence, we need to perform barcoding sequence data analysis more efficiently. Otherwise, biodiversity research will be increasingly constrained by the computational resources available.

To alleviate this scalability challenge we introduce a novel tool, which we call raxtax, and demonstrate that it is at least as accurate as the widely-used existing tools SINTAX, IDTAXA, RDP, and BayesANT. Furthermore, we demonstrate that raxtax is 2.7 to 100 faster in comparison to the competing tools listed.

raxtax achieves high accuracy in conjunction with computational efficiency via a *k*-mer based matching approach. That is, we formulate sequence similarity as follows: Compute the expected number of matching *k*-mers between the reference sequence and a random sampling of the *k*-mers of a query sequence. The key insight is that if a query sequence is more similar to a reference sequence, the number of expected matching *k*-mers will be higher. Other tools have used analogous sampling techniques to great effect [e.g. MetaCache in the context of metagenomic studies (Müller *et al.* 2017, Wood *et al.* 2019)]. Here, instead of sampling *k*-mers, we devise an analytical solution. With this reformulation of the problem, we can derive closed analytical solutions that allow for computing the exact probability that a given reference sequence is (among) the best matches for a random sample of query sequence *k*-mers. Given a set of DNA reference sequences (each with a taxonomic annotation), raxtax computes the best-match probabilities for each anonymous query sequence, and reports the best matching lineages with their per-rank confidence scores by aggregating these probabilities at each taxonomic rank (clade). Finally, we also use these per-rank confidence scores to compute uncertainty scores for each assignment of a query to a lineage. Each of these quantities and their interpretations are discussed in Section 2. raxtax is available as open-source code and pre-compiled binaries at https://github.com/noahares/raxtax under a CC-NC-BY-SA license.

## 2 Materials and methods

Given a sequence S (consisting of characters from the set {A,C,G,T}), a *k*-mer is a sub-sequence S[i..i+k],i∈[|S|−k] of length *k*. The set of *k*-mers, *Q*, associated with S includes all unique *k*-mers of S. For our current implementation of raxtax, we fix k:=8 to allow for some computational optimizations (see Section 3.1), but in principle the method can be adapted to any *k*.

Strictly matching *all k*-mers of each query sequence against *all* reference sequences is not only time and memory intensive, but also highly sensitive to sequencing errors (Ma *et al.* 2002). On the other hand, only matching a small random sample of *k*-mers does not constitute an appropriate solution either. In particular, if the reference sequences are highly similar and/or share a large fraction of *k*-mers, numerous repetitions with small random samples will be required to distinguish between plausible assignments and therefore increase run-times. Instead, we use a combinatorial approach for selecting a random subset of *k*-mers from the query to match against the reference. This allows to obtain accurate results while being computationally efficient at the same time.

Assume that we are given the set of all *k*-mers *Q* which have been extracted from a query sequence and that we intend to match them against a set of reference sequences $D=\{D_{1},\ldots ,D_{n}\}$. For each $D_{i}$ there exists a corresponding set of all *k*-mers contained therein, denoted by $K_{i}$. Let $K=\{K_{1},\ldots ,K_{n}\}$ be the set of all *k*-mer sets. To find the best matching $K_{i}$ for a given *Q*, we need to identify the $K_{i}$ which maximizes the expected number of matches from a random sampling of *t k*-mers from *Q*. We label this sample as $S_{t}(Q)$. Define $P_{i}$ as the probability that the reference *k*-mer set $K_{i}$ has the most *k*-mers in common with a random sampling of *Q*, or more formally:


(1) $$K_{i}\cap S_{t}(Q)\geq K_{j}\cap S_{t}(Q)\;\forall K_{j}\in K.$$

Our method for computing this probability is described in Sections 3.3 and 3.4.

Define the probability that a reference *k*-mer set $K_{i}$ has *m* matching *k*-mers with $S_{t}(Q)$ as


(2) $$p_{i}(m):=P(|K_{i}\cap S_{t}(Q)|=m),$$

which is a probability mass function (PMF). Using this definition, we can now compute the cumulative mass function (CMF) by marginalizing over the possible match sizes that are indexed by *l*. Then, we take the product over the other references indexed by *j* to compute the probability of no other reference having more than *m* matches,


(3) $$c_{i}(m):=\prod_{j\neq i}\left(\sum_{l\leq m}p_{j}(l)\right).$$

The probability that $K_{i}$ is among the best matches, given a sample size *t* then is


(4) $$\begin{array}{c} P_{i}:=\sum_{m\leq t}p_{i}(m)c_{i}(m). \end{array}$$

Additionally, we normalize the values in *P* via the *L*1 norm in order to compute *confidence (scores)*. This operation simplifies the subsequent confidence accumulation at different taxonomic ranks. As a result, the reported values are not, strictly speaking, probabilities. Instead, they report the confidence regarding the relative ranking of reference for matching a query.

Given a clade *B* of the reference taxonomy, we define the confidence of *B* being among the best matches relative to other clades of the same rank as


(5) $$L(B):=\sum_{D_{i}\in B}\frac{P_{i}}{||P|{|}_{1}}.$$

**Figure 1.**
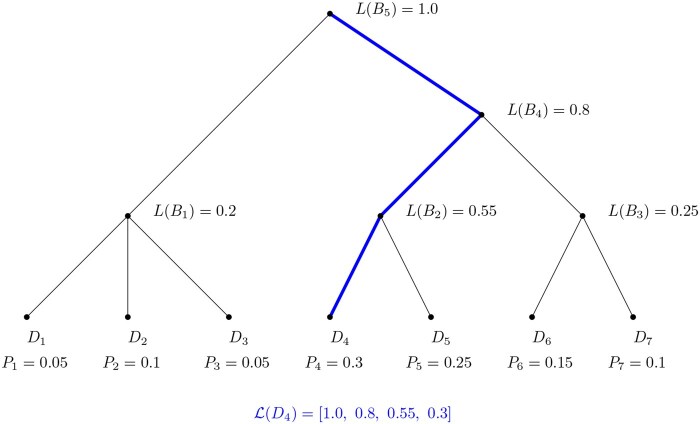
A simple lineage showing how a L-vector is constructed. The contributors to $L(D_{4})$ are highlighted.

To simplify the notation, we define $L(D_{i})$ as the lineage confidence vector for reference sequence $D_{i}$. $L(D_{i})$ is a sequence of L(·) values for the taxonomic lineage, where $A_{i}$ is a series of nested partitions (clades) of the reference sequences ($D_{i}=A_{0}\subseteq \ldots \subseteq A_{i}\subseteq \ldots \subseteq D$). An example lineage tree with a lineage confidence vector for a reference sequence $D_{4}$ is shown in Fig. 1.

### 2.1 Uncertainty scores

The per-rank confidence values L(·) that we compute with raxtax will be biased by the taxonomic distribution of reference sequences in the database. Because the values at high-level ranks are the sum over all per-sequence values within those ranks, interpreting a confidence value of 0.5 requires knowledge about the relative frequency of that clade in the reference database. For instance, consider the case that one family represents 50% of the database. In this case, by chance alone, a substantial proportion of the total confidence score will be assigned to reference sequences in this over-represented family. Therefore, to better interpret the confidence values relative to the reference database properties, we report two additional uncertainty scores.

Let $\bar{P}:=\left(\frac{1}{n},\ldots ,\frac{1}{n}\right)$ be the *expected* confidence vector for a sequence that is highly dissimilar (i.e. *k*-mer set intersections will be of approximately the same size) to all reference sequences. In analogy to using *L* for *P* values (Equation (5)), we define $\bar{L}$ as the *expected* confidence of obtaining a higher-level rank assignment based on $\bar{P}$. This means that the expected values of higher-level ranks represent the potential database bias. We will use these values to derive an uncertainty score for the global (*per-sequence*) and local (*per-rank*) assignment signals, i.e. the deviation of the observed confidence values from the expected values based on the reference database bias.

The *local assignment signal*


(6) $$s_{l}(D_{i}):={\left\|\frac{L(D_{i})}{{\left\|L(D_{i})\right\|}_{1}}-\frac{\bar{L}(D_{i})}{{\left\|\bar{L}(D_{i})\right\|}_{1}}\right\|}_{2},$$

quantifies the uncertainty in $L(D_{i})$ as the Euclidean distance between the computed and expected per-rank confidence values (with normalization). Analogously, we define the *global assignment signal*


(7) $$s_{g}:={\left\|P-\bar{P}\right\|}_{2}$$

to quantify the reference sequence level confidence scores as the Euclidean distance between the computed and expected per-sequence confidence values. We describe how to interpret and use the local and global assignment signals in Supplement 5, available as supplementary data at *Bioinformatics* online.

## 3 Implementation

raxtax is written in Rust (compiled with version 1.76) and is parallelized over the query sequences using the rayon library (https://github.com/rayon-rs/rayon). In this section, we describe the algorithmic techniques and data structures we use to optimize raxtax.

### 3.1 Calculating intersection sizes

To compute the match scores for all query-reference pairs, we need to compute the intersection of the two *k*-mer sets. Because computing intersection sizes accounts for at least half of the processing time of a query it is important to optimize them. A naïve implementation requires computing O(nm) intersections, where *n* is the number of query sequences, and *m* is the number of reference sequences. The best case run time for a sorted set intersection of sets *A* and *B* is O(min(|A|,|B|)) via a linear scan when A⊆B.

While there exist numerous fast set intersection algorithms (Schlegel *et al.* 2011), most pairs of *k*-mer sets satisfy |A∩B|≪min(|A|,|B|). Hence, it will be more efficient to ask which reference sequences contain a specific *k*-mer and store these results in a lookup table. This lookup table is computed once for all *k*-mers and reference sequences and is query-independent. It can therefore be saved for any analyses that use the same reference database. Given this lookup table, we simply perform a lookup of the *k*-mers in the query sequence to compute the intersection of a query-reference pair. Thereby, we reduce the work for one query-reference pair from O(min(|A|,|B|)) to O(|A∩B|), where *A* and *B* are the respective *k*-mer sets.

Because we discard *k*-mers that include gaps and ambiguous characters, they can be represented in a memory-efficient manner by only using two bits per DNA character. By setting k:=8, we can thus uniquely store an 8-mer in a 16-bit unsigned integer (u16) by using its corresponding bit representation. While parsing the reference sequences, we create a lookup table that for each 8-mer (represented as a u16) holds a sorted list of reference sequences that contain it. When extracting the *k*-mers from a query sequence later-on, we can use this lookup table to rapidly identify those reference sequences that contain each query sequence *k*-mer. This allows to efficiently create an array of intersection sizes with all reference sequences on demand.

### 3.2 Post-order lineage tree

**Figure 2.**
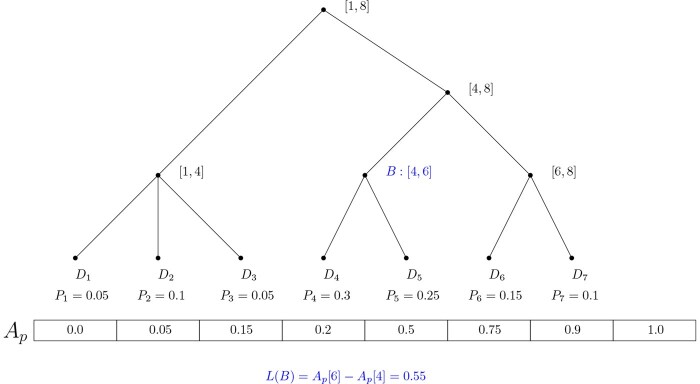
A simple lineage showing the prefix sum $A_{p}$ and inner nodes indices. An example for node *B* is given.

The core of raxtax is a multi-furcating tree data structure that reflects the entire lineage tree of the reference sequence set *D*. For each query, we create a new array *A* of size |*D*| to hold the normalized confidence scores from Equation (4). The indices of *A* correspond to the leaves of the tree in post-order. Each inner node *B* of the tree also stores an integer pair (*a*, *b*) that contains the index interval of *A* that belongs to the rank associated with this node. After computing the confidence scores as described in Section 2 and storing them in *A*, we compute their prefix sum $A_{p}$. To subsequently determine the clade confidence score L(B) (see Equation (5)) for any clade *B* of the tree, we calculate it via $A_{p}[b]-A_{p}[a]$ as can be seen Fig. 2.

We stop computing further L(·) values when the confidence of a node drops below a threshold of 0.005 to avoid an unnecessary evaluation of the entire tree. Thereby, we only report relevant lineages.

### 3.3 The probability of exactly *m* matching *k*-mers

We defined the PMF $p_{i}(m)$ of a reference *k*-mer set $K_{i}$ having exactly *m* out of *t* matches in Equation (2). If we expand this, we obtain


(8) $$p_{i}(m):=\frac{\left(\begin{array}{c} |Q\cap K_{i}|+m-1\\ m \end{array}\right)\left(\begin{array}{c} |K_{i}|-|Q\cap K_{i}|+(t-m)-1\\ t-m \end{array}\right)}{\left(\begin{array}{c} |Q|+t-1\\ t \end{array}\right)}.$$

Note that for a given query, the divisor is fixed and only depends on the size of the *k*-mer set *Q* of the query sequence and *t*, i.e. the number of *k*-mers to be sampled. Also note that we need to calculate the numerator for each m≤t with *m* being the only variable. By utilizing the equivalence


(9) $$\left(\begin{array}{c} n+1\\ k+1 \end{array}\right)=\left(\begin{array}{c} n\\ k \end{array}\right)\frac{n+1}{k+1},$$

we can iteratively compute both binomial coefficients in the numerator by only using a single multiplication and division per each value of *m*.

### 3.4 Caching PMF and CMF values

We define


(10) $$C(m):=\prod_{j\in [n]}\sum_{l\in [m]}p_{j}(l),$$

where the inner sum is the CMF over $p_{j}$ for a reference *k*-mer set $K_{j}$. Therefore, C(m) is the product over all CMFs for some match count *m*. Given this definition, we can compute


(11) $$P_{i}=\sum_{m\in [t]}p_{i}(m)\frac{C(m)}{c_{i}(m)}$$

via 2*t* additional operations. Computing all PMF and CMF values has complexity $O(|D|t^{2})$. Using Equation (11) decreases the additional time complexity for computing *P* from $O(|D{|}^{2}t)$ to O(|D|t). That is, the computation of best-match probabilities is reduced by a factor of |*D*|. For all but the smallest reference databases, t≪|D|, so this caching substantially accelerates the computation.

### 3.5 Improving runtime for repeated execution with the same reference sequences

The lineage tree (cf. Section 3.2) and *k*-mer-to-sequence mapping (cf. Section 3.1) are independent of any queries and can therefore be shared between runs using the same reference sequences. To this end, we save the reference database in a binary file using bincode (https://github.com/bincode-org/bincode) which conducts encoding and decoding via a tiny binary serialization strategy. This file can initially be generated and then used for further queries at a later time. Often, this saves a substantial amount of time on reference databases that comprise a large amount of sequences and/or long sequences. In our experiments with the BOLD database (Ratnasingham and Hebert 2007), using the binary file created by bincode is two times faster than parsing the original input.

## 4 Experimental evaluation

We use three datasets from widely-used databases: UNITE ITS (Abarenkov *et al.* 2024), Greengenes 16S (McDonald *et al.* 2024), BOLD COX1 (Ratnasingham and Hebert 2007). In each dataset, we only retained entries with complete taxonomic information and also removed duplicate sequences (Table 1). Further details about the databases can be found in Supplement 1, available as supplementary data at *Bioinformatics* online. We conducted additional experiments with real-world Operational Taxonomic units (OTUs) from a large experiment of meta-barcoding data from insect traps across Germany (Buchner *et al.* 2025) and evaluated the fraction of equivalent identifications between the different tools. Among the tools, raxtax showed the highest agreement, with 97.66% of its classifications shared with at least one other tool. This evaluation can be found in Supplement 6, available as supplementary data at *Bioinformatics* online.

**Table 1.** Databases.

Database	UNITE	Greengenes	BOLD
Highest taxonomic rank	Fungi	Bacteria	Arthropoda
No. of sequences	47 154	187 329	1 254 059
No. of unique species	31 479	629	136 622

We compare raxtax (v1.2.2) against four other taxonomic assignment tools: SINTAX (vsearch v2.28.1), RDP (v2.14–0), IDTAXA (DECIPHER v3.2.0), and BayesANT (v1.0).

The experiments were conducted on a 2-socket machine with 2x Intel(R) Xeon Platinum 8260 CPUs @ 2.40 GHz with 48 physical cores (96 threads) in total. Each tool was executed with 48 threads (except RDP, which can only use two threads) to avoid hyper-threading, unless stated otherwise.

### 4.1 Cross-validation benchmarks

To evaluate raxtax, we performed a 10-fold cross-validation with random splits of the databases into 90% reference and 10% query sequences, and calculated the $F_{1}$ score to assess the accuracy (TP = True Positives, MC = Missclassified, FN = False Negatives, FP = False Positives) at different taxonomic ranks


(12) $$\text{Recall}=\frac{\text{TP}}{\text{TP}+\text{MC}+\text{FN}}$$

(13) $$\text{Precision}=\frac{\text{TP}}{\text{TP}+\text{FP}}$$

(14) $$F_{1}=2*\frac{\text{Recall}*\text{Precision}}{\text{Recall}+\text{Precision}}$$

**Figure 3.**
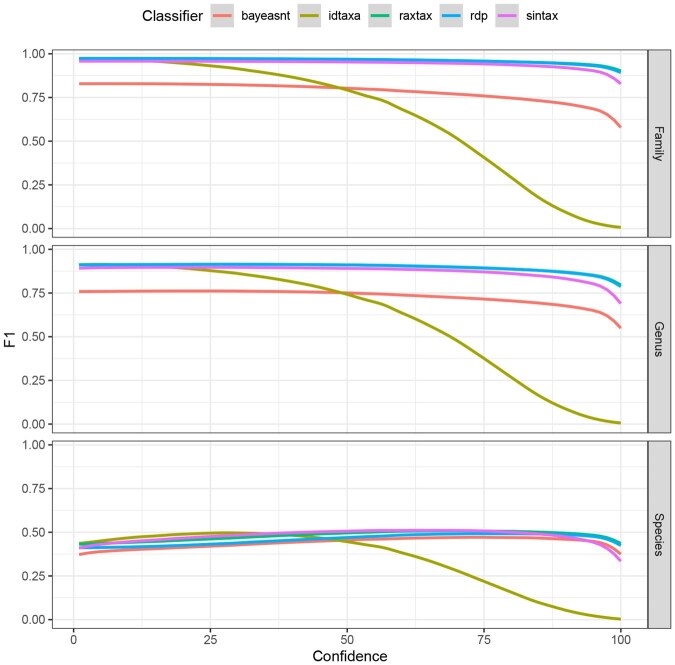
$F_{1}$ scores (*y*-axis) for classification of UNITE sequences at the family, genus, and species level (top to bottom) where the reported confidence exceeds the confidence cutoff (*x*-axis).

**Figure 4.**
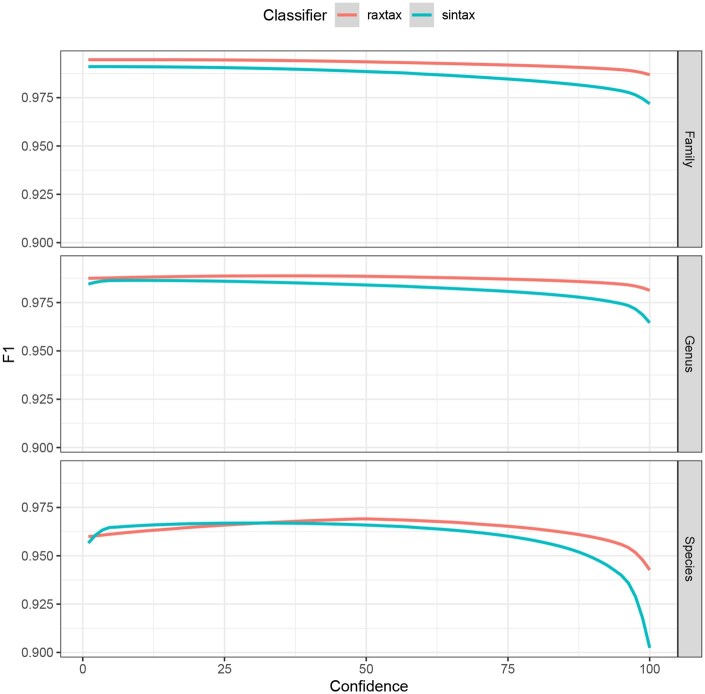
$F_{1}$ scores (*y*-axis) for classification of BOLD sequences at the family, genus, and species level (top to bottom) where the reported confidence exceeds the confidence cutoff (*x*-axis).

Each tool provides a confidence score for the result of each query assignment and for each taxonomic rank that ranges between 0 and 100. We evaluated our algorithm against the competing tools by setting a continuous confidence cutoff thresholds that labels all results below the respective cutoff as “not classified”. In this context, “misclassified” means that a sequence was assigned to the wrong lineage with a confidence score higher than the threshold. We then calculate the $F_{1}$ score for each confidence cutoff value (Figs 3 and 4).

Figure 3 shows that on the UNITE database, raxtax, RDP, and SINTAX perform equally well at all taxonomic levels. Further, raxtax and RDP are indistinguishable at the family and genus level. IDTAXA was developed to circumvent over-classification. Hence, once the confidence threshold approaches values of 25–50 the computed $F_{1}$ scores rapidly decline as a consequence of this conservative approach. BayesANT is only competitive at the species level.

For sequences from the BOLD database (Fig. 4) only raxtax and SINTAX finished all 10 cross-validations within the 48 h time limit, so we compare only their $F_{1}$ scores. Partial results including RDP and IDTAXA can be found in Supplement 3, available as supplementary data at *Bioinformatics* online. The raxtax  $F_{1}$ score is consistently better at the family and genus level. At species level, the difference is statistically significant under the Wilcoxon signed-rank test with the alternative hypothesis that raxtax has higher $F_{1}$ scores, and the matched pairs rank-biserial correlation (RBC, effect size) is large (Wilcoxon 1992) ($p=6.3680\times 10^{-77}$, RBC: 0.6763). The standardized mean difference (Cohen’s *d*, (Cohen, 1988)) is medium sized (d=0.5549), indicating that while the $F_{1}$ scores of raxtax are consistently higher, the differences are only marginal. In general, both tools perform exceptionally well at classifying these sequences. However, as we show in the following sections, raxtax is 2.7 times faster than SINTAX for the comparatively large BOLD database and exhibits growing speedups as we simultaneously increase the number of query *and* reference sequences.

Results for the Greengenes database can be found in Supplement 2, available as supplementary data at *Bioinformatics* online.

### 4.2 Performance benchmarks

We measured the runtime and memory requirements of each tool for a single test (i.e. one out of the 10 cross-validations) on each dataset (Table 2). We set a time limit of 48 h—a common job time limit on clusters—to accommodate for tradeoffs between accuracy and time requirements. On the BOLD dataset, only raxtax, SINTAX, and RDP completed within the memory and time limits. We observed that RDP and BayesANT require more resources as a function of the unique species number in the reference, while SINTAX and IDTAXA performance depends on the number of query and reference sequences. Datasets will continue to grow over time, both, in terms of the species diversity they cover, and the number of query as well as reference sequences they contain. Hence, we expect that the computational resource requirements of some of the tools we tested might prohibit their future deployment.

**Table 2.** Time and memory requirements for a single cross-validation.

Database	UNITE		Greengenes		BOLD^a^	
Resource	*T* (s)	*M* (GiB)	*T* (s)	*M* (GiB)	*T* (m)	*M* (GiB)
raxtax	2	0.53	237	3.17	13	9.93
SINTAX	12	0.13	94	1.25	35	3.78
RDP	399	10.99	166	0.61	1302	50.52
IDTAXA	2202	3.69	3643	5.40	^b^	
BayesANT	320	3.52	217	9.61	^c^	

a Run-times on BOLD are in minutes instead of seconds.

b Exceeded time limit (48 h).

c *R* error (attempt to make table with ≥2^31^ elements).

### 4.3 Snapshot benchmark

**Figure 5.**
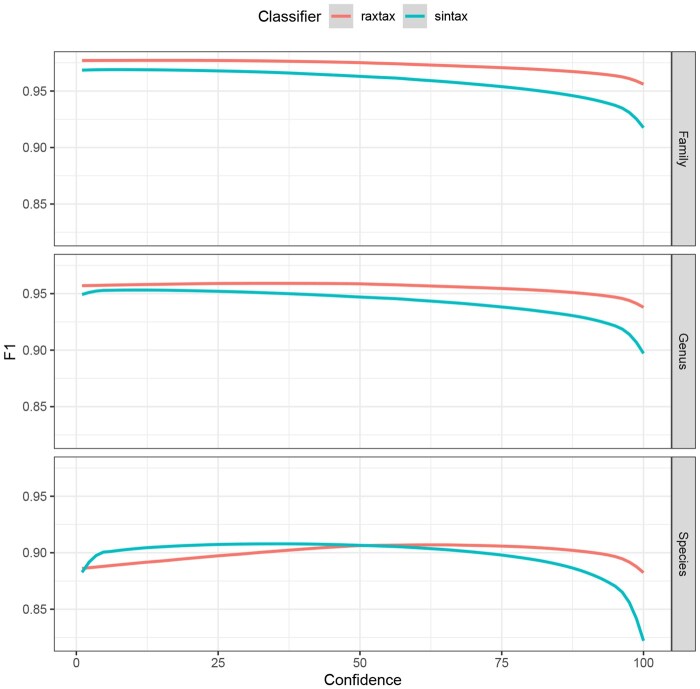
$F_{1}$ scores (*y*-axis) for the classification of BOLD snapshots at the family, genus, and species level (top to bottom) where the reported confidence exceeds the confidence cutoff (*x*-axis).

In order to validate our algorithm via a more realistic setting, we used two different BOLD database snapshots that were generated 11 months apart from each other. We taxonomically classified the sequences that were added during these 11 months by treating them as query sequences and subsequently compared the inferred annotation results with the respective “true” taxonomic annotation. Given the data volume of this analysis, only raxtax and SINTAX were able to terminate within the 48 h time limit using 48 threads. The $F_{1}$ scores are shown in Fig. 5. As for the 10-fold cross-validation on the BOLD database (Fig. 4), raxtax and SINTAX are equally accurate. raxtax again outperforms SINTAX at the family and genus level. However, the difference at species level is not statistically significant in this test. Both the effect size (Wilcoxon with two-sided alternative, p=0.7635, RBC: 0.0349) and the standardized mean difference (d=0.1427) are small. Here, raxtax is 5.62 times faster than SINTAX.

### 4.4 Time and memory scaling

**Figure 6.**
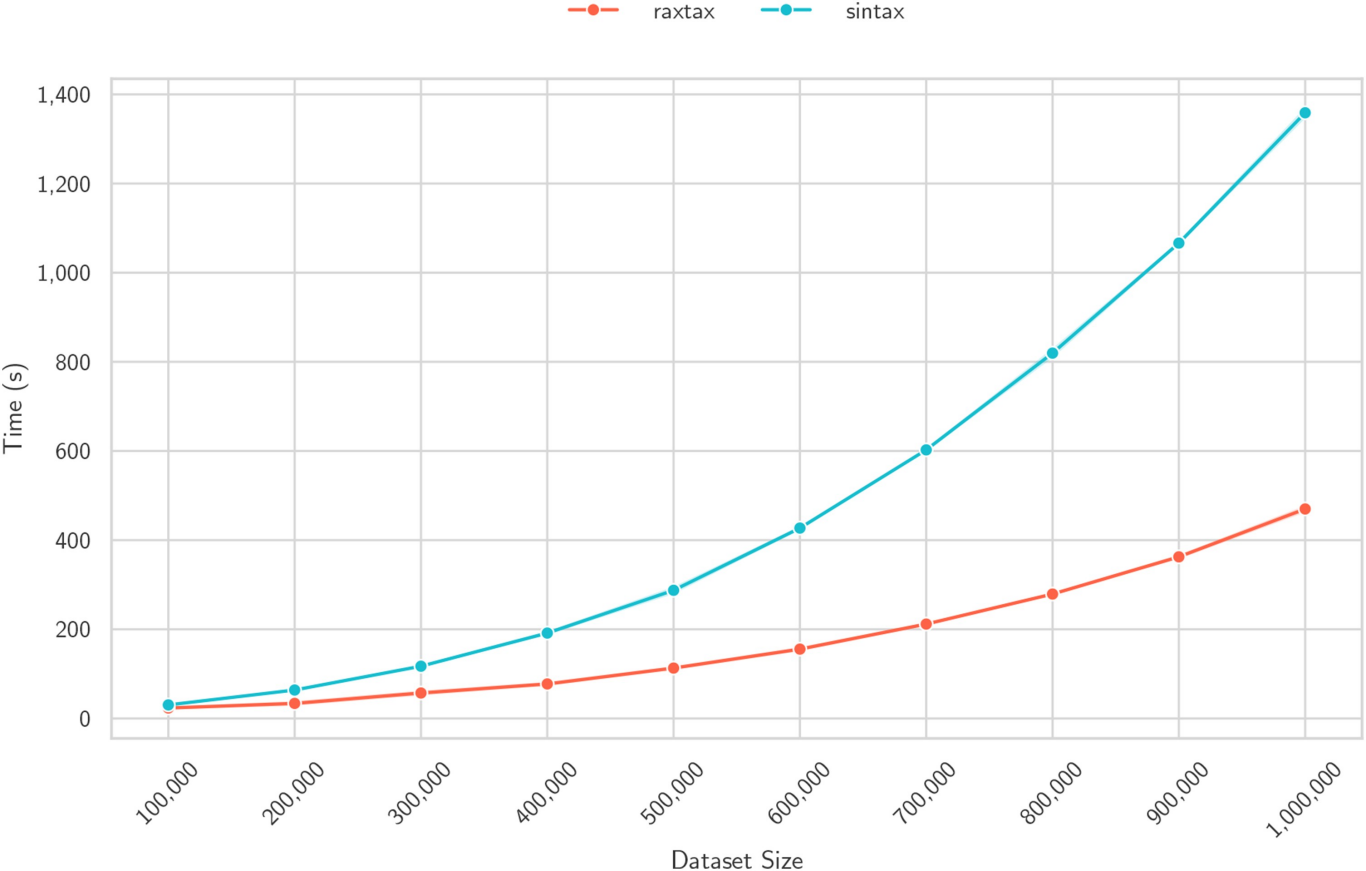
Time (*y*-axis) for classification of BOLD samples of different sizes (*x*-axis). 90% of the sample is the reference, the remaining 10% are the queries (three random samples per sample size).

Figure 6 shows super-linear runtime scaling for both tools when we simultaneously increase the number of reference *and* query sequences. The number of threads for both tools is again fixed to 48. raxtax clearly scales better when we increase the number of query and reference sequences. Going from 100 000 to 1 000 000 total sequences the speedup over SINTAX increases from 1.3 to 2.9.

**Figure 7.**
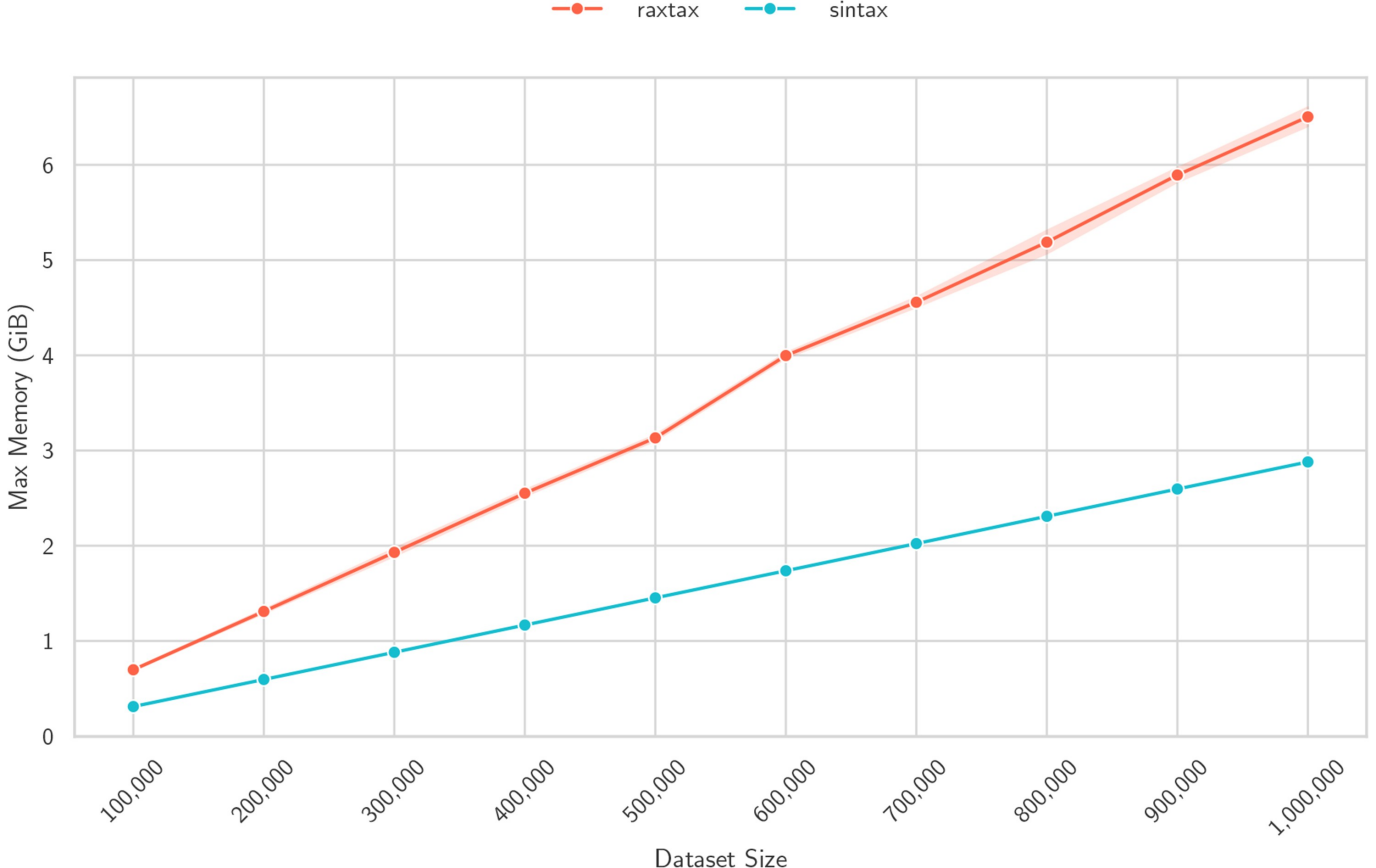
Maximum memory usage in GiB (*y*-axis) for classification of BOLD samples of different sizes (*x*-axis). 90% of the sample is the reference, the remaining 10% are the queries (three random samples per sample size).

In terms of memory requirements (Fig. 7), both tools exhibit a linear memory scaling as the total dataset size (no. of query and reference sequences) increases. The main memory requirements of raxtax (and presumably SINTAX as well) are dominated by the data structures that hold the reference database. Hence, this linear scaling is expected. SINTAX exhibits lower memory requirements and better scaling when we increase the total number of sequences. However, even for the whole BOLD database raxtax’s memory requirements remain below 10 GiB (see Table 2), so we argue that this is a favorable resource tradeoff for using raxtax because of faster run times. BOLD currently contains the largest amount of barcodes for meta-barcoding projects and the memory consumption of raxtax increases by roughly 1 GB per 150 000 sequences. Therefore, we believe that raxtax can be used without issues on mid-range laptops for the foreseeable future.

**Figure 8.**
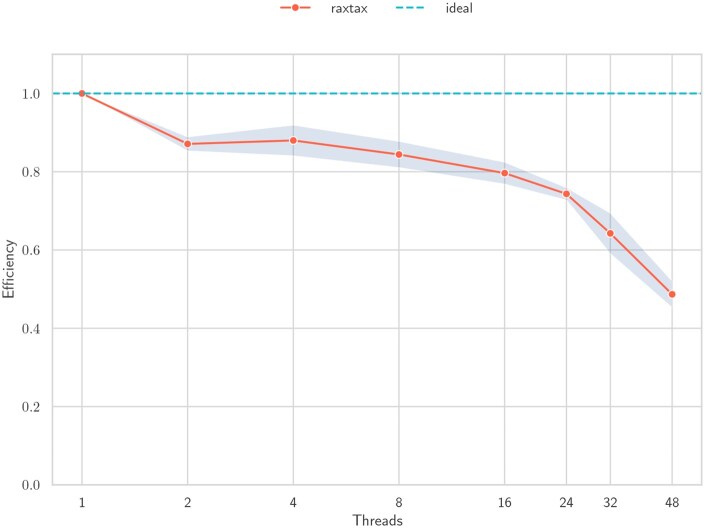
Strong self-relative efficiency and standard deviation (*y*-axis) over increasing thread numbers (*x*-axis). The reference database size is fixed at 450 000 sequences with 50 000 queries (90–10 split), and we randomly sample five times.

We also measure strong parallel efficiency for raxtax for a varying number of threads on samples from the BOLD database. Figure 8 shows a gradual decline in parallel efficiency from two threads (efficiency: 0.87) to 24 threads (efficiency: 0.74) compared to the baseline with one thread. Thereafter, parallel efficiency continues to rapidly deteriorate with increasing number of threads.

We use explicit thread-pinning to avoid executing threads on the same physical core and schedule threads to the same socket if possible (up to 24 threads). Therefore, the more rapid decline in parallel efficiency at 32 and 48 threads is to be expected as cross-socket communication overhead is introduced. See Supplement 4, available as supplementary data at *Bioinformatics* online, for more details and discussion about thread-pinning and weak parallel speedup.

## 5 Conclusion

We have presented a novel analytical approach for classifying unlabeled sequences based on *k*-mer matching and derive the equations of our match scoring function for determining the best matching taxonomic lineage. Our method also introduces two additional uncertainty scores that are sensitive to an unbalanced distribution of ranks in the reference database and thereby provide users more context for drawing informed conclusions. We implemented this approach in raxtax as open-source software. Further, we conducted a thorough code optimization to ensure that the tool is fast and efficient. An extensive evaluation of raxtax in conjunction with a comparison to existing tools demonstrates that we attain better or equivalent classification accuracy based on $F_{1}$ scores. Further, raxtax can handle the ever-increasing dataset sizes in taxonomic classification and can efficiently use all available computational resources on modern hardware. We argue that the increased memory requirements compared to SINTAX are an acceptable tradeoff for the reduced run-times. In the future, we aim to deploy raxtax as part of a comprehensive meta-barcoding pipeline for real-world queries and adapt our approach in order to apply it beyond short barcoding sequences. Finally, we intend to investigate the design of a distributed memory parallelization.

## Supplementary Material

btaf620_Supplementary_Data

## Data Availability

This tool is available as source code and pre-compiled binaries at https://github.com/noahares/raxtax under a CC-NC-BY-SA license. The scripts and summary metrics used in our analyses are available at https://github.com/noahares/raxtax_paper_scripts. The source code, sequence data and summarized results of the analyses are available at https://doi.org/10.5281/zenodo.15057027.

## References

[btaf620-B1] Abarenkov K , Nilsson RH, Larsson K-H et al The UNITE database for molecular identification and taxonomic communication of fungi and other eukaryotes: sequences, taxa and classifications reconsidered. Nucleic Acids Res 2024;52:D791–97. 10.1093/nar/gkad1039 37953409 PMC10767974

[btaf620-B2] Buchner D , Sinclair JS, Ayasse M et al Upscaling biodiversity monitoring: metabarcoding estimates 31,846 insect species from malaise traps across Germany. Mol Ecol Resour 2025;25:e14023. 39364584 10.1111/1755-0998.14023 PMC11646302

[btaf620-B3] Cohen J. Statistical Power Analysis for the Behavioral Sciences. New York, NY, USA: Routledge, 1988.

[btaf620-B4] Edgar RC. Sintax: a simple non-Bayesian taxonomy classifier for 16s and its sequences. biorxiv, 074161, 10.1101/07416, 2016, preprint: not peer reviewed.

[btaf620-B5] Elbrecht V , Taberlet P, Dejean T et al Testing the potential of a ribosomal 16s marker for DNA metabarcoding of insects. PeerJ 2016;4:e1966. 27114891 10.7717/peerj.1966 PMC4841222

[btaf620-B6] Hebert PD , Cywinska A, Ball SL et al Biological identifications through DNA barcodes. Proc R Soc Lond B 2003a;270:313–21. 10.1098/rspb.2002.2218 PMC1691236 12614582

[btaf620-B7] Hebert PD , Ratnasingham S, De Waard JR. Barcoding animal life: cytochrome c oxidase subunit 1 divergences among closely related species. Proc R Soc Lond B: Biol Sci 2003b;270(suppl_1):S96–9. 10.1098/rsbl.2003.0025 PMC1698023 12952648

[btaf620-B8] Janssen PH. Identifying the dominant soil bacterial taxa in libraries of 16s rRNA and 16s rRNA genes. Appl Environ Microbiol 2006;72:1719–28. 16517615 10.1128/AEM.72.3.1719-1728.2006 PMC1393246

[btaf620-B9] La Salle J , Williams KJ, Moritz C. Biodiversity analysis in the digital era. Phil Trans R Soc B 2016;371:20150337. 27481789 10.1098/rstb.2015.0337 PMC4971189

[btaf620-B10] Liu X , Zhang L, Hong S. Global biodiversity research during 1900–2009: a bibliometric analysis. Biodivers Conserv 2011;20:807–26.

[btaf620-B11] Ma B , Tromp J, Li M. PatternHunter: faster and more sensitive homology search. Bioinformatics 2002;18:440–5. 11934743 10.1093/bioinformatics/18.3.440

[btaf620-B12] McDonald D , Jiang Y, Balaban M et al Greengenes2 unifies microbial data in a single reference tree. Nat Biotechnol 2024;42:715–8. 10.1038/s41587-023-01845-1 37500913 PMC10818020

[btaf620-B13] Murali A , Bhargava A, Wright ES. IDTAXA: a novel approach for accurate taxonomic classification of microbiome sequences. Microbiome 2018;6:140–14. 30092815 10.1186/s40168-018-0521-5 PMC6085705

[btaf620-B14] Müller A , Hundt C, Hildebrandt A et al MetaCache: context-aware classification of metagenomic reads using minhashing. Bioinformatics 2017;33:3740–8. 10.1093/bioinformatics/btx520 28961782

[btaf620-B15] Ratnasingham S , Hebert PDN. bold: the barcode of life data system. Mol Ecol Notes 2007;7:355–64. 18784790 10.1111/j.1471-8286.2007.01678.x PMC1890991

[btaf620-B16] Ratnasingham S , Wei C, Chan D. BOLD v4: a centralized bioinformatics platform for DNA-based biodiversity data. Mol Biol 2024;2744:403–41. 10.1007/978-1-0716-3581-0_26 38683334

[btaf620-B17] Schlegel B , Willhalm T, Lehner W. Fast sorted-set intersection using SIMD instructions. Adms@ VLDB 2011;1:1–8.

[btaf620-B18] Schoch CL , Seifert KA, Huhndorf S et al; Fungal Barcoding Consortium. Nuclear ribosomal internal transcribed spacer (ITS) region as a universal DNA barcode marker for fungi. Proc Natl Acad Sci 2012;109:6241–6. 22454494 10.1073/pnas.1117018109 PMC3341068

[btaf620-B20] Wang Q , Garrity GM, Tiedje JM et al Naive Bayesian classifier for rapid assignment of rRNA sequences into the new bacterial taxonomy. Appl Environ Microbiol 2007;73:5261–7. 17586664 10.1128/AEM.00062-07 PMC1950982

[btaf620-B21] Ward RD , Zemlak TS, Innes BH et al DNA barcoding Australia’s fish species. Phil Trans R Soc B 2005;360:1847–57. 16214743 10.1098/rstb.2005.1716 PMC1609232

[btaf620-B22] Wetterstrand KA. DNA Sequencing Costs: Data from the NHGRI Genome Sequencing Program (GSP). National Human Genome Research Institute, 2019. Available at: www.genome.gov/sequencingcostsdata (20 March 2025, date last accessed).

[btaf620-B23] Wilcoxon F. Individual comparisons by ranking methods. In: Breakthroughs in Statistics: Methodology and Distribution. New York, NY, USA: Springer, 1992, 196–202.

[btaf620-B24] Wood DE , Lu J, Langmead B. Improved metagenomic analysis with Kraken 2. Genome Biol 2019;20:257. 10.1186/s13059-019-1891-0 31779668 PMC6883579

[btaf620-B25] Yang R-H , Su J-H, Shang J-J et al Evaluation of the ribosomal DNA internal transcribed spacer (ITS), specifically ITS1 and ITS2, for the analysis of fungal diversity by deep sequencing. PLoS One 2018;13:e0206428. 30359454 10.1371/journal.pone.0206428 PMC6201957

[btaf620-B26] Zito A , Rigon T, Dunson DB. Inferring taxonomic placement from DNA barcoding aiding in discovery of new taxa. Methods Ecol Evol 2023;14:529–42.

