# Supplementary material for: raxtax: a k-mer-based non-Bayesian taxonomic classifier
Source: Bioinformatics. 2025 Nov 19;41(12):btaf620. doi: 10.1093/bioinformatics/btaf620 (PMC12677947; doi:10.1093/bioinformatics/btaf620)
Supplement: btaf620_Supplementary_Data [file btaf620_supplementary_data.pdf]

raxtax: A k-mer-based non-Bayesian Taxonomic Classifier Supplement

Noah A. Wahl[1], Georgios Koutsovoulos[1], Ben Bettisworth[1], and Alexandros Stamatakis[1,2,3]

[1]Biodiversity Computing Group, Institute of Computer Science, Foundation for Research and Technology Hellas, 100 Nikolaou Plastira, 70013 Heraklion, Crete, Greece
[2]Computational Molecular Evolution Group, Heidelberg Institute for Theoretical Studies, Schloss-Wolfsbrunnenweg 35, 69118 Heidelberg, Baden-Württemberg, Germany
[3]Institute for Theoretical Informatics, Karlsruhe Institute of Technology, Kaiserstraße 12, 76131 Karlsruhe, Baden-Württemberg, Germany

1 Detailed Database Specifications

The UNITE, Greengenes and BOLD databases are split into 90% reference sequences and 10% query sequences for all experiments. The BOLD Snapshot consists of reference sequences added up to 2023-09-29. The query sequences contain all sequences added in the following 11 months up to 2024-08-23. The number of query and reference sequences are listed in Table 2 below:

Database	Reference Sequences	Query Sequences
UNITE	38,653	4295
Greengenes	168,596	18,733
BOLD	1,128,653	125,406
BOLD Snapshot	2,038,078	419,821

Table 1: Databases

2 Greengenes Cross Validation Benchmarks

The sequences of the Greengenes database were by far the easiest to classify. Except IDTAXA, all tools perform almost perfectly on family and genus level. Only on species level differences become apparent: While BayesANT and RDP experience slight drop-offs once the confidence cutoff approaches 100, `raxtax` maintains the highest F_1-score throughout all cutoff values. SINTAX has overall lower F_1-scores on species level and experiences an even stronger drop-off than BayesANT and RDP once the cutoff is beyond 90.

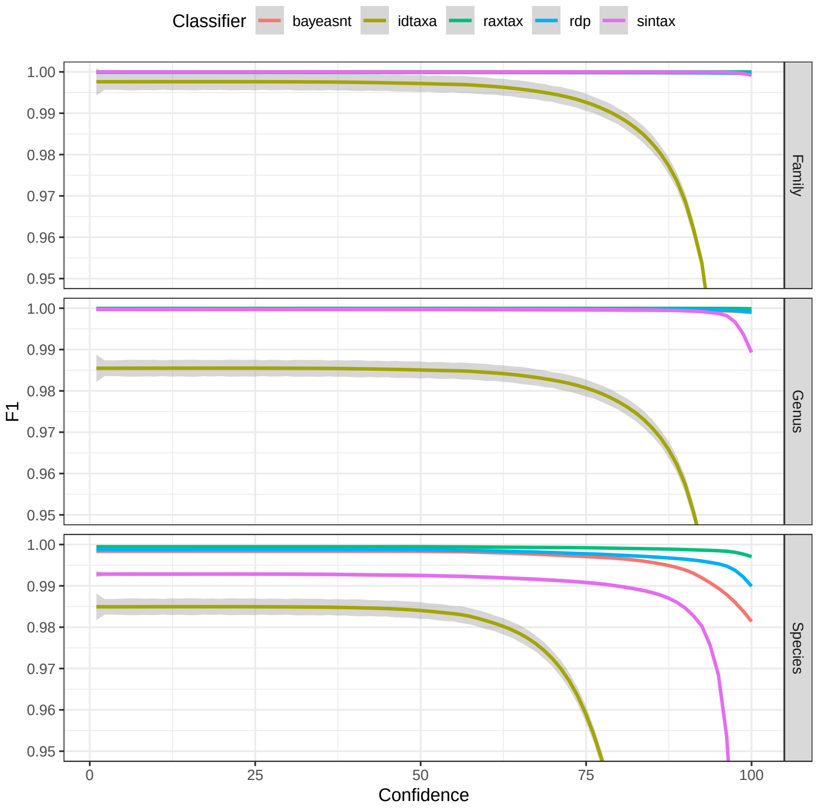

Figure 1: F_1 scores (y-axis) for classification of Greengenes sequences at the family, genus, and species level (top to bottom) where the reported confidence exceeds the confidence cut-off (x-axis).

3 Incomplete BOLD Cross Validation Benchmarks

In Section 4.1 of the main paper we excluded RDP and IDTAXA from the results because they did not finish all 10 cross validations. Here we include them in the comparison of F_1 scores for **one** of the 10 cross validation runs. As with the UNITE and Greengenes databases, RDP and `raxtax` perform equally well. IDTAXA once again cannot compete once the cutoff exceeds 50 due to its conservative approach.

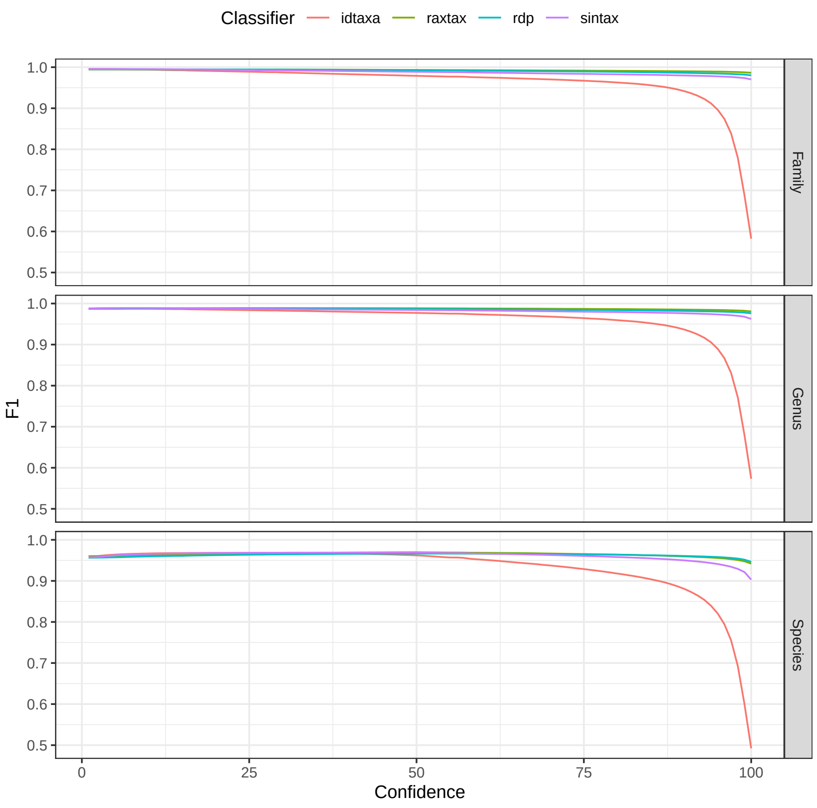

Figure 2: F_1 scores (y-axis) for classification of BOLD sequences at the family, genus, and species level (top to bottom) where the reported confidence exceeds the confidence cut-off (x-axis).

4 Weak Speedup and Thread-pinning Evaluation

In Figure 3 we show the results of weak parallel speedup experiments. As `raxtax` conducts increasing work (i.e., additional queries) relative to the number of threads, we observe a gradual decrease in parallel speedup compared to the baseline with 1 thread. Overall, using thread-pinning (TP) and scheduling threads to the same socket if possible (up to 24 threads) shows better results compared to leaving the assignment of threads to the operating system. The super-linear speedup at 2 threads for `raxtax` without thread-pinning can be explained by cache coherence, as large data structures are mutually read by different threads. We attribute the gradual decline in parallel efficiency to cache contention for the L3-cache, which is shared by all physical cores of a socket. We note that the standard deviation of speedups reaches up to 0.13, indicating that the choice of reference and query sequences heavily influences the run time. In this setup, the ”difficulty” of the random queries for a different number of threads can change significantly, because we add 2,000 additional queries per thread, so these results need to be interpreted with caution. Generally, we recommend using thread-pinning when running `raxtax`.

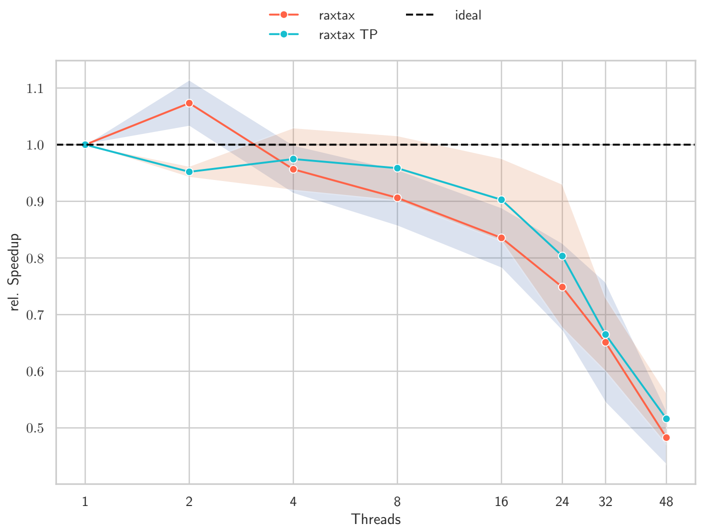

Figure 3: Weak self-relative speedup (y-axis) and standard deviation over increasing thread numbers (x-axis). The reference database size is fixed at 250,000 sequences, that are randomly sampled 5 times. For each additional thread a further 2,000 query sequences are included. We compare `raxtax` without thread-pinning (red) and `raxtax` with thread-pinning (cyan).

5 Using Local and Global Assignment Signals

A challenge when interpreting classification scores is that they are affected by the database composition. Thus, exclusively reporting the best match may obfuscate close runner-ups that need to also be considered. We address this challenge via our *local* assignment signal. It quantifies to which extent the assignment deviates from the expected scores based on the respective clade sizes in the given reference database. Results with a *large* local assignment signal are therefore more trustworthy. In contrast, results with a *small* local assignment signal may indicate that the confidences are inflated by an over-representation of that specific clade in the reference database. Note that for highly unbalanced databases, the maximum local assignment signal will be very low. We deliberately do not normalize the score to $[0, 1]$ to increase user awareness regarding this bias.

The *global* assignment score is identical for all results computed for a query. It indicates to which degree the results can be distinguished from random matches at the sequence level. If the global assignment signal is large and roughly corresponds to the species level confidence score, the result may be interpreted as being unambiguous. In this case the query will have but a few matches with other reference sequences. If the global assignment score is small compared to the species score, there will be an increased number of matches with other reference sequences. In this case, users should inspect the results to verify or discard the assignment.

As these signals constitute a novel approach to quantify assignment confidence, we currently cannot yet provide reasonable thresholds for what should be considered as "large" or "small". We further strongly discourage users from using them as primary scores for assignment evaluation. We recommend using large signals for justifying assignment results and small signals for critically evaluating assignment results.

6 Benchmarking tools with real-world data

We have downloaded the Operational Taxonomic units (OTUs) from a large insect metabarcoding data experiment with insect traps across Germany [1]. We classified a total of 61,148 OTUs using all tools against a BOLD reference database containing 2,159,792 unique sequences. BayesANT yielded the same R error as in Table 2 of the main paper and was therefore excluded from the comparisons. Subsequently, we selected those 31,270 OTUs for which at least one of the tools inferred a confidence score of at least 0.7. We then compared these OTUs at the species level to identify areas of agreement and discrepancy among the tools and visualised the results with UpSetR ([2, 3]). All four tools agree on the species-level classification for 74.48% of the OTUs, while the remaining classifications yield different intersecting sets (Figure 4). Among the tools, `raxtax` showed the highest agreement, with at least one other tool for 97.66% of its classifications, while RDP showed the lowest agreement with 87.05%.

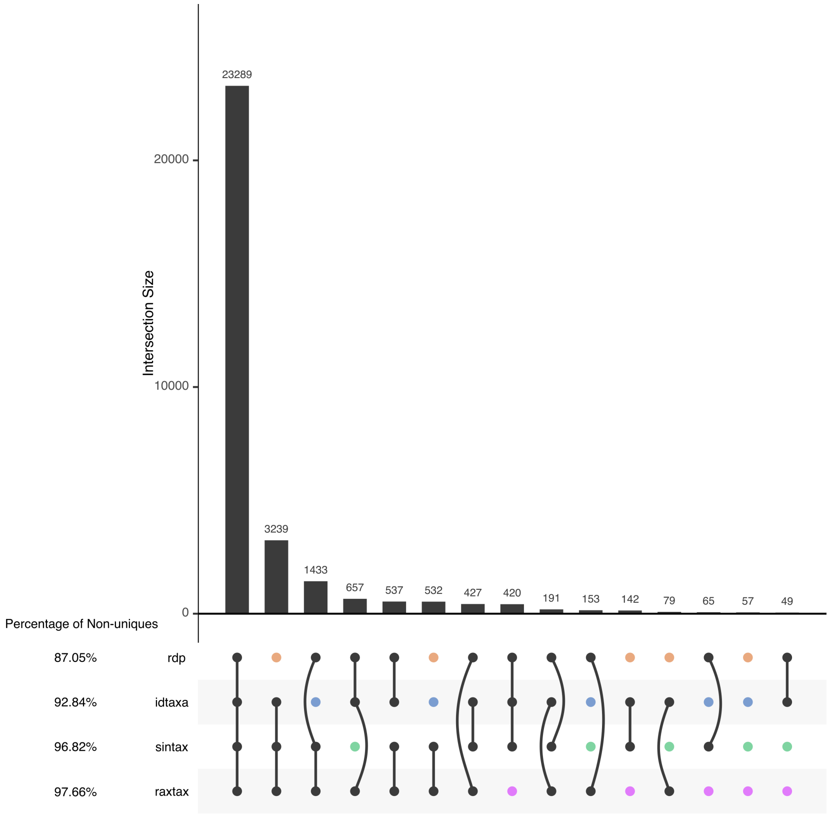

Figure 4: UpSet plot of shared classifications between the different tools.

Time and memory requirements are similar to the results in Table 2 of the main manuscript. `raxtax` is significantly faster than all other tools (12.75x faster than SINTAX), but requires twice as much memory as SINTAX.

Tool	Time (hh:mm:ss)	Memory (GiB)
`raxtax`	00:04:16	13.39
SINTAX	01:01:49	6.45
RDP	09:34:31	113.74
IDTAXA	*93:20:16	33.15
BayesANT	†	

*exceeded time limit (48h)

†R error (attempt to make table with >= 2^{31} elements)

Table 2: Time and memory requirements for queries with real-world data.

References

[1] Dominik Buchner, James S Sinclair, Manfred Ayasse, Arne J Beermann, Jörn Buse, Frank Dziock, Julian Enss, Mark Frenzel, Thomas Hörren, Yuanheng Li, et al. Upscaling biodiversity monitoring: Metabarcoding estimates 31,846 insect species from malaise traps across germany. *Molecular Ecology Resources*, 25(1):e14023, 2025.

[2] Alexander Lex, Nils Gehlenborg, Hendrik Strobelt, Romain Vuillemot, and Hanspeter Pfister. Upset: visualization of intersecting sets. *IEEE transactions on visualization and computer graphics*, 20(12):1983–1992, 2014.

[3] Jake R Conway, Alexander Lex, and Nils Gehlenborg. Upsetr: an r package for the visualization of intersecting sets and their properties. *Bioinformatics*, 33(18):2938–2940, 2017.
